# Depth Refuge and the Impacts of SCUBA Spearfishing on Coral Reef Fishes

**DOI:** 10.1371/journal.pone.0092628

**Published:** 2014-03-24

**Authors:** Steven J. Lindfield, Jennifer L. McIlwain, Euan S. Harvey

**Affiliations:** 1 The UWA Oceans Institute and School of Plant Biology, Faculty of Natural and Agricultural Sciences, The University of Western Australia, Crawley, Western Australia, Australia; 2 Department of Environment and Agriculture, Curtin University, Bentley, Western Australia, Australia; Aristotle University of Thessaloniki, Greece

## Abstract

In recent decades, spearfishing with SCUBA has emerged as an efficient method for targeting reef fish in deeper waters. However, deeper waters are increasingly recognised as a potential source of refuge that may help sustain fishery resources. We used a combination of historical catch data over a 20-year time period and fishery-independent surveys to investigate the effects of SCUBA spearfishing on coral reef fish populations in the southern Mariana Islands. Two jurisdictions were studied; Guam, where SCUBA spearfishing is practiced, and the nearby Commonwealth of Northern Mariana Islands (CNMI), where SCUBA spearfishing has been banned since 2003. Fishery-independent data were collected using baited remote underwater stereo-video systems (stereo-BRUVs) stratified by depth, marine protected area status and jurisdiction. Herbivores (primary consumers) dominated spearfishing catches, with parrotfish (scarines) and surgeonfish/unicornfish (acanthurids) the main groups harvested. However, the large, endangered humphead wrasse (*Cheilinus undulatus*) was the main species by weight landed by SCUBA spearfishers. SCUBA spearfishing was associated with declining size of scarines over time and catches shifting from a dominance of large parrotfishes to a mixed assemblage with increasing proportions of acanthurids. Comparisons between Guam and the nearby CNMI revealed differences in the assemblage of fished species and also greater size of scarines and acanthurids in deep water where SCUBA fishing is banned. These results suggest that SCUBA spearfishing impacts reef fish populations and that the restriction of this fishing method will ensure refuge for fish populations in deeper waters. We recommend a ban on SCUBA spearfishing to preserve or aid the recovery of large, functionally important coral reef species and to improve the sustainability of coral reef fisheries.

## Introduction

Spearfishing is an important method for harvesting reef-associated fish worldwide. Like other fishing methods, spearfishing has undergone significant modifications through time, evolving from handmade spears and basic skin-diving equipment to high-powered guns, underwater lights and the utilisation of self-contained underwater breathing apparatus (SCUBA) to maximise catches. Spear guns are a highly selective fishing gear, yet often the method is used non-selectively for commercial profit or to target species with life histories that cannot sustain high levels of fishing pressure [1]. Although spearfishing has been regarded as an unsustainable fishing technique when unregulated [1]–[3], management regulations such as protecting certain species or introducing size and catch limits could work positively with the inherently high selectivity of the method [4], [5].

It is increasingly recognised that management is required to ensure sustainable spearfishing catches, especially in the Pacific Islands where human populations are increasing and spearfishing is often the primary method for subsistence fishing [1], [6]–[8]. Apart from direct consumption needs, spearfishing is also commercially valuable, with 75% of marketed reef fish in Micronesia sourced from night-time spearfishing [9]. Targeted species are most efficiently caught at night when they are sleeping on the reef and easily visible to spearfishers. When combined with access to commercial markets and no catch restrictions, spearfishing at night can quickly deplete inshore fish resources [3], [10]. Spearfishers also harvest herbivorous species on coral reefs [6], [11]. However, herbivorous fish play an important functional role in regulating algal growth on coral reefs [12], [13] and effective ecosystem-based management may warrant restrictions on the use of spear guns when coral reefs are dominated by algae [6], [8]. Despite spearfishing presenting a number of concerns for management, Gillet and Moy [1] concluded in their comprehensive assessment of spearfishing in the Pacific Islands that the single most important management measure was to prohibit the use SCUBA for spearfishing and the effective enforcement of such bans.

SCUBA spearfishing remains legal in various regions around the world, from temperate locations such as the south-eastern Pacific (Chile, Peru, Ecuador) and some states of Australia, to numerous tropical locations in the Indo-Pacific [1], [2]. Guam is a Pacific Island where SCUBA spearfishing has been practiced for over 25 years and contributes to the commercial reef-fish fishery [9], [11], [14]. Despite declining reef fishery catches in Guam and proposed legislation to ban the method since the early 1990s [14]–[16], such management has yet to be implemented. Many Pacific Island countries banned the use of SCUBA for spearfishing soon after its inception due to concerns regarding efficiency, fishery declines, the fact that it is non-traditional and that it conflicts with snorkel fishermen and underwater tourism [1], [17], [18]. For example, in American Samoa during 1994, the rapid change from subsistence-based, snorkel spearfishing to commercial SCUBA spearfishing resulted in parrotfish catches increasing 15-fold [17], [19]. Rather than waiting for long-term evidence of the impacts, fishery managers applied the precautionary approach and the practice was banned in 2001. At the time, large parrotfishes and humphead wrasse (*Cheilinus undulatus*) were absent or rare at heavily fished reefs [17]. More recently, surveys of American Samoa's coral reefs revealed that populations of key reef species are in a stable state and parrotfish populations are showing signs of recovery [20].

The potential for deeper waters to protect species from natural or anthropogenic disturbances is increasingly recognised as pertinent to marine conservation planning and resource management [21]–[24]. Many coral reef fish, especially mobile targeted species, are wide-ranging in their depth distribution along the reef slope [25]. Yet certain fishing methods, particularly breath-hold spearfishing, have obvious depth limitations. It is therefore assumed that a proportion of the fish population can obtain refuge in deeper water (Fig. S1). Referred to as “depth refuge”, only two studies have explored the validity of this theory for coral reef fish [26], [27]. Protection afforded by deeper waters could allow depth generalist species to repopulate shallower waters, as demonstrated previously with abalone [28] and corals [29]. Depth may also provide effects of protection similar to those of marine protected areas (MPAs), where the biomass, density and size of fish can increase compared to nearby fished areas [30]. In this scenario it is plausible for adult fish to migrate vertically from deeper waters to the heavily fished shallow waters, rather than just horizontally along the reef.

Fishery-independent surveys often provide only a snapshot of the fish community in time and space, making it difficult to infer historical changes in fish stock structure. Fortunately, a comprehensive and regular series of creel surveys was initiated throughout the U.S. flag-associated islands in the Pacific during the 1980s, providing a means to examine historical catches in Guam [16]. The aim of this study was to combine these historical catch data with fishery-independent surveys to investigate the impact of SCUBA spearfishing in Guam and the Commonwealth of the Northern Mariana Islands (CNMI). Specifically we set out to: 1) analyse creel survey data to determine which reef fish species dominate the spearfishing catch and how catch composition and fish size have changed over time; and 2) conduct fishery-independent surveys to detect potential impacts of SCUBA spearfishing on the assemblage structure, biomass and lengths of fished species between depths and across locations with different levels of fishing pressure and management.

## Methods

### Ethics Statement

Ethics approvals were not required for observational studies of fish at the time of this study. All research activities complied with regulations of the Guam Department of Agriculture's Division of Aquatic and Wildlife Resources and the CNMI Division of Fish and Wildlife. Permission or permits were not required to access the study areas as there was no capture, handling, collection or harassment of fish or wildlife including endangered or protected species.

### Study Area

**Figure 1:**
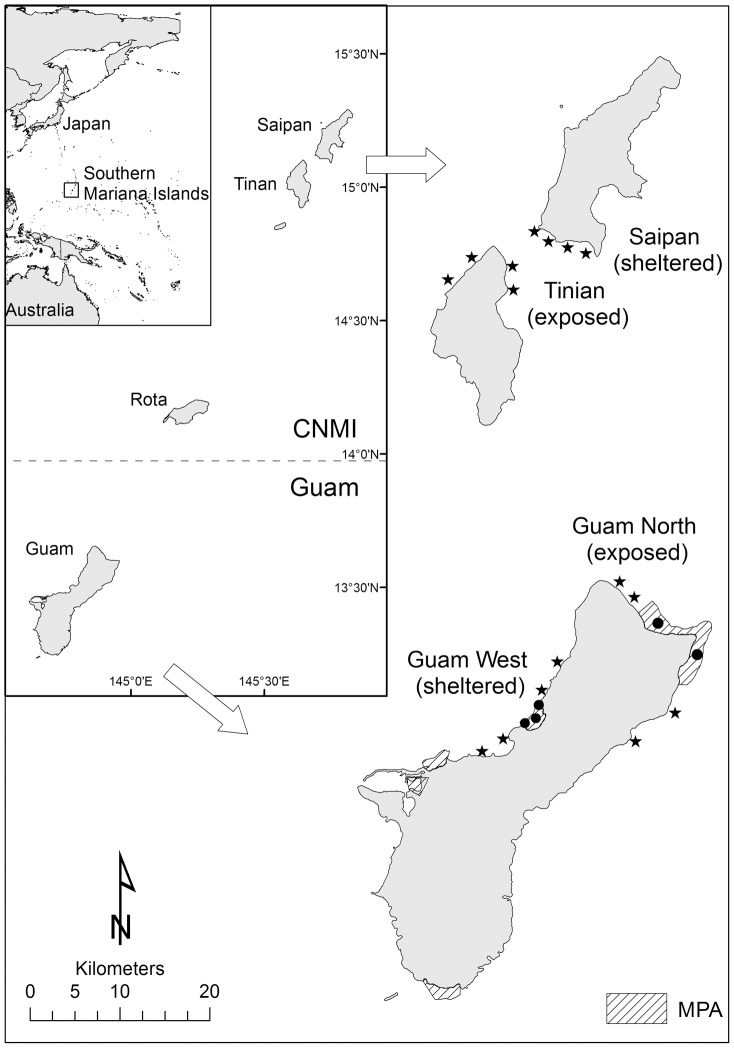
Study locations in the southern Mariana Islands. Sample sites denoted as black dots (inside MPAs) and stars (fished areas). Marine protected areas (MPAs) on Guam are indicated by diagonal shading.

Guam and the CNMI are two jurisdictions in the Pacific that allow a case study for assessing the impact of SCUBA spearfishing (Fig. 1). Similar to many Pacific Islands, the introduction of a cash-based economy along with increasing population size, development and tourism, has placed strain on the sustainable use of natural resources such as reef fish [15], [31]. The largest and southernmost of the Mariana Islands, Guam (13.50° N, 144.8° E) is an unincorporated territory of the United States with a human population of over 159 000 (2010 census). Located to the north of Guam, the Commonwealth of the Northern Mariana Islands (CNMI) is an island archipelago with a population of approximately 54 000 people, of which 90% live on the island of Saipan (15.18° N, 144.75° E). Tinian (15.00° N, 145.63° E) is located 5 km south-west of Saipan with a human population of 3 136 (2010 census).

Guam and the CNMI both have MPAs, yet outside these areas there are no fishing regulations on size, quantity of catch, protected species or seasonal closures, and no licensing is required for the capture or commercial sale of fish. The CNMI has additional gear-based regulations that include a ban on the use of gillnets, and since 2003, a ban on SCUBA spearfishing [18]. Marine protected areas in Guam are not strictly no-take (allowing limited fishing from shore and trolling for pelagic species), however spearfishing is prohibited within their boundaries [32]. The Tumon Bay MPA at the sheltered Guam West location (Fig. 1) covers an area of 4.52 km^2^ and is close to the main population centre [32]. Due to the proximity to the main population centre, enforcement of this MPA is high. Pati Point MPA at the exposed Guam North location covers an area of 20 km^2^, but effective enforcement is limited as it is located furthest from any boat ramp in the remote northern part of the island (Fig. 1).

### Historical catch data

Since 1983 the Guam Department of Aquatic and Wildlife Resources (DAWR), in collaboration with the Western Pacific Fisheries Information Network (WPacFIN) has collected a comprehensive series of catch estimates through regular creel surveys (fishermen interviews) in Guam. These creel survey interviews are conducted on four randomly selected days each month through boat-based and shore-based surveys which document the fishing method used and the fish species caught and their size. For the first two years of surveys, sampling did not include the night-time catch of SCUBA spearfishing [14]. Then after 2005 many SCUBA spearfishers refused to participate in the survey (as it remains voluntary), hence the landing data for this fishery after 2006 are severely underestimated and not representative of the total catch [33]. For these reasons we limited our data analysis to the 20 years from 1986 to 2005. These creel data were used to compare catch composition between SCUBA and snorkel spearfishing, changes in assemblage structure, and the average length and contribution to catch for the dominant fish species and families over time.

### Fishery-independent survey

#### Experimental design

We used fishery-independent surveys to assess the impacts of spearfishing on reef fish populations under different management scenarios. Wave exposure was incorporated into our study design because it is known to affect the biomass of herbivorous fish in Micronesia [34]. We surveyed two locations (sheltered and exposed) at each jurisdiction (Guam and CNMI) (Fig. 1). Sampling sites on Guam were placed either side of MPA reference sites. A total of 21 sites were sampled, four sites at each of the four fished locations, plus three sites within the MPA at Guam West and two sites within the MPA at Guam North. The majority of sites were selected a-priori from existing multibeam bathymetry data [35] to include the following criteria; at least 30 m depth and high complexity reef. Because the habitat in northern Saipan was unsuitable at 30 m (e.g. low complexity Halimeda algal reefs) and the island has a large lagoon system on the west coast, we chose sites on the sheltered southern coast of Saipan and the exposed north coast of Tinian, which featured a similar habitat to the Guam locations. Two depth categories were sampled at each site; 10 m and 30 m. These depths were chosen to distinguish differences in the fish assemblages due to snorkel and SCUBA spearfishing methods. SCUBA spearfishers regularly dive to depths of 30 m or deeper [14] while snorkel spearfishers frequently dive to 10 m, but rarely to depths of 30 m.

#### Sampling technique

Baited remote underwater stereo-video systems (stereo-BRUVs) were used for several reasons. First, diver survey methods are inefficient at depths of 30 m because of limitations on repetitive scientific diving. Second, we observed fishery targeted species to be wary of divers when conducting preliminary surveys by underwater visual census. Third, cost–benefit analyses have shown stereo-BRUVs to be more cost-effective at detecting change in the biomass of herbivorous fish in coral reef habitats than diver surveys [36]. Finally, the use of bait provides greater statistical power than using un-baited remote video stations by attracting greater numbers of predatory and scavenging species without decreasing the abundances of herbivorous species [37]. The stereo-BRUVs used in this study were the same as described by Langlois et al [36] but used high definition Sony CX-7 camcorders. For bait we used one kg of cut and crushed Pacific saury (*Cololabis saira*). Each stereo-BRUV system was deployed for 60 minutes as commonly performed by other studies [26], [36], [37]. We deployed five stereo-BRUVs at each site and replicates were separated by at least 150 m. A total of 210 stereo-BRUVs were deployed between the 1^st^ July and 29^th^ October 2010.

#### Video analysis

We analysed stereo-BRUVs footage using EventMeasure-Stereo software [38]. Abundance was estimated using the MaxN method (as reviewed by Cappo et al. [39]). The stereo configuration and calibration of the video cameras allowed us to accurately measure fish length (fork length) and distance from the cameras [40]–[42]. To ensure accuracy of the length measurements while accurately identifying and counting as many fish as possible, we used the following guidelines; small-bodied individuals up to 100 mm length were sampled within 4 m of the cameras, fish to 500 mm were sampled to 8 m distance and larger fish were sampled to a maximum distance up to 10 m from the cameras.

#### Biomass calculation

Biomass was calculated from length measurements using length-weight relationships developed from the Guam creel survey data that recorded accurate fish lengths and weights. Length-weight regression values *a* and *b* were calculated from fork length (mm) and weight (g) for 159 targeted species. We used these values to calculate the weight for each individual fish using the allometric relationship: weight (grams)  =  *a* x length (mm)*^b^*. For individual fish that could not be measured (e.g. being obscured from one of the camera views) we used the average length for that species from the site where it occurred.

#### Grouping of fish

Species were placed into one of four functional groups following Sandin and Williams [43]. Primary consumers (herbivores and detritivores) were a focus for analysis as they are the main functional group caught by spearfishers and are not commonly caught using other methods, such as line fishing [3], [6], [8]. We also analysed fished species as a group, which consisted of the top 100 species that contributed to total biomass from each spearfishing method in addition to similar species expected to be highly targeted. The large roving piscivores, dogtooth tuna (*Gymnosarda unicolour*) and barracuda (*Sphyraena barracuda*) were excluded from univariate analysis as less than 8 individuals were observed yet these species dominated biomass estimates when present. Juveniles of all species (<100 mm) were not included in the analysis as they are not targeted by spearfishers and would bias the average length calculations.

### Statistical analysis

The percentage contribution of biomass was based on standardised data as the intensity of creel survey interview data was not consistent between years or methods. To illustrate changes in assemblage structure over time, we created a multivariate dataset of fish species that were present in at least five years of the survey and contributed greater than 1% of total SCUBA spearfishing catch. Data were analysed with PRIMER 6 statistical software [44] using square-root transformed data and the Bray-Curtis resemblance matrix. To visualise patterns, we used non-metric multidimensional scaling (nMDS) [45] with each data point representing a year and subsequent years linked using a trajectory line. A Pearson's correlation coefficient of greater than +0.3 was used to determine species that correlated with the clustering of data points.

Three-way permutational multivariate analysis of variance (PERMANOVA) [46] was used to test for differences between factors *MPA Status* or *Jurisdiction* (fixed: MPA vs fished or Guam vs CNMI), *Depth* (fixed: shallow vs deep) and *Site* (random, nested in MPA status x Depth or Jurisdiction x Depth). A Modified Gower (log base 10) transformation was used to create the resemblance matrix and standardise the range of biomass values as estimates varied by several orders of magnitude between species [47]. *P*-values were obtained using permutation tests (9999 permutations) for each individual term in the model. Constrained canonical analysis of principal coordinates (CAP) [48] was then used to investigate differences in assemblage structure between these factors. The number of axes (m) was manually chosen by plotting the residual sum of squares and choosing the first significant drop in relation to the other values. Spearman rank correlation value of greater than +0.45 was used to show potential relationships between individual species and the canonical axes.

To test the univariate hypothesis that the biomass of fished species and primary consumers differed between depths and levels of fishing pressure, we used general linear model analysis of variance (ANOVA). Prior to performing ANOVAs, homogeneity of variance was tested using Levene's tests and data were square-root transformed where necessary. The two Guam locations (Guam West and Guam North) were analysed separately because they cover different exposures and accessibility to fishers. The 3-way experimental design to test for main effects and interaction terms followed that described for PERMANOVA. We analysed the lengths of scarines and acanthurids using the same methods, but pooled data across sites. Significant interaction terms for fixed effects were examined further using Tukey's simultaneous tests for pairwise multiple comparisons.

## Results

### Historical catch data

#### Catch composition

**Figure 2:**
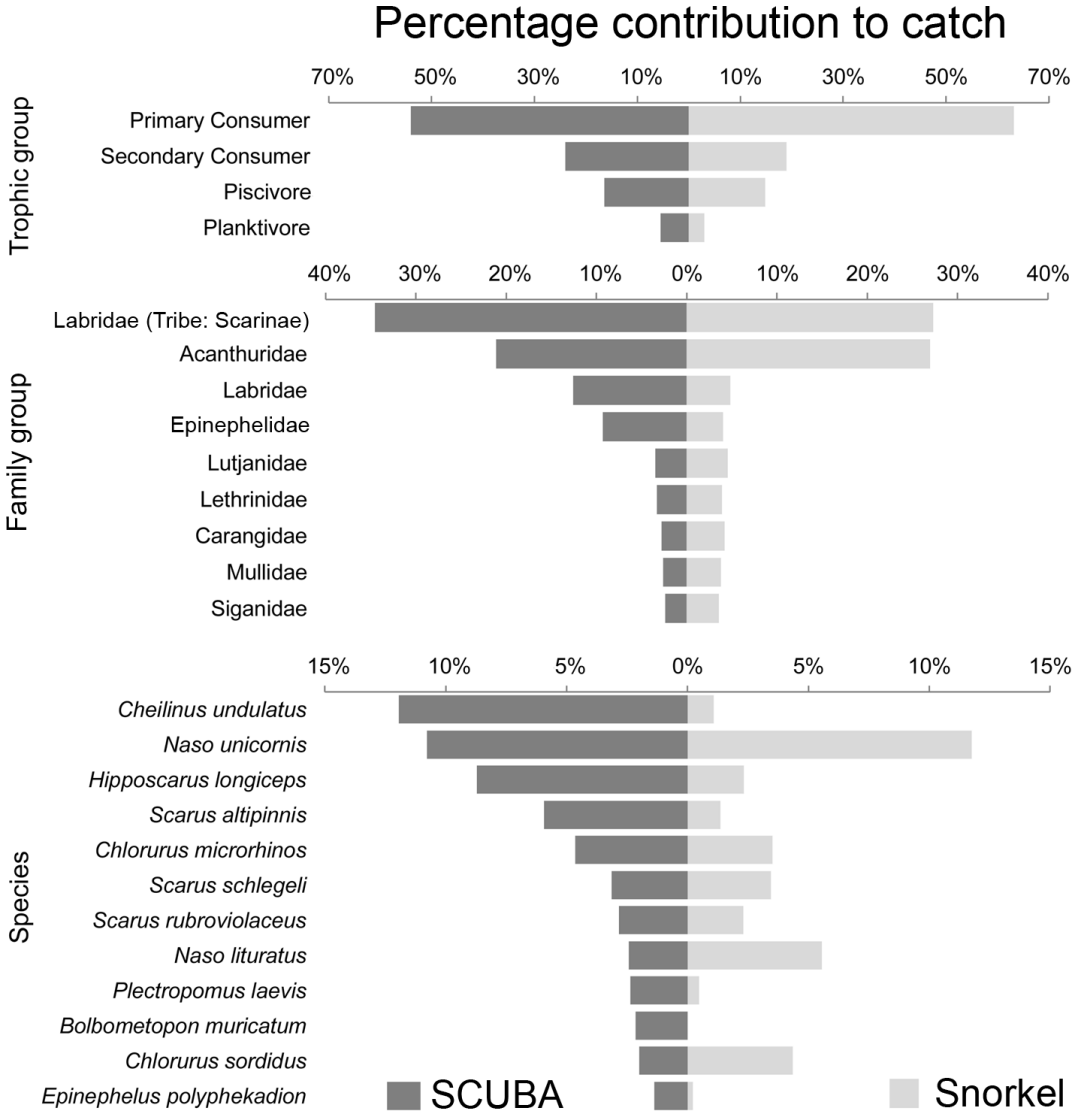
Percentage contribution of spearfishing catches in Guam. Biomass summed across years from 1985–2005 for trophic group, family groups and species caught by SCUBA (left side dark bars) or snorkel spearfishing (right side light bars).

Primary consumers (herbivores and detritivores) were the main trophic group contributing to spearfishing catch in Guam (Fig. 2). Parrotfish (Labridae; tribe Scarinae) were the main group caught by SCUBA spearfishing (35% of catch) followed by the surgeonfish, tangs and unicornfish (Acanthuridae) (21% of catch). SCUBA spearfishers also caught greater proportions of wrasse (Labridae) and grouper (Epinephelidae) compared to snorkel spearfishing (Fig. 2). The single species that contributed the greatest biomass to SCUBA spearfishing catch was the humphead wrasse (*Cheilinus undulatus*). Overall, 95% of the total spearfishing catch of *C. undulatus* was caught with SCUBA. The bluespine unicornfish (*Naso unicornis*) was the next greatest contributor to SCUBA spearfishing catch, followed by the parrotfishes *Hipposcarus longiceps* and *Scarus altipinnis*, which were both more dominant in the SCUBA catch compared to the snorkel catch. SCUBA spearfishing also caught three large-bodied reef fish that were rarely caught by snorkel spearfishers: bumphead parrotfish (*Bolbometopon muricatum*); camouflage grouper (*Epinephelus polyphekadion*) and the blacksaddled coral grouper (*Plectropomus laevis*).

#### Changes over time

**Figure 3:**
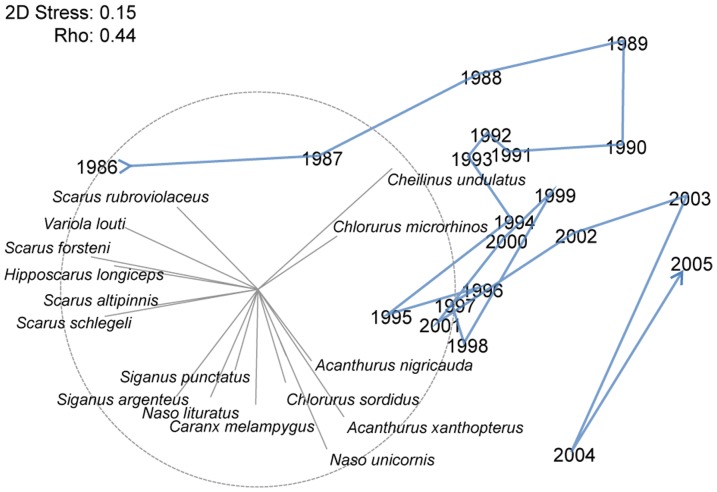
nMDS plot of the relative biomass contribution of species regularly caught by SCUBA spearfishing. Subsequent years between 1985 and 2005 are linked by a trajectory line and species correlations are indicated by the length and direction of vectors.

The species composition of the SCUBA spearfishing catch changed over the 20 year time period (Fig. 3, Rho: p<0.05). Between 1986 and 2005 the catch shifted from a dominance of large bodied parrotfishes (*Scarus forsteni*, *Scarus rubroviolaceus, Scarus schlegeli, H. longiceps*) and the grouper (*Variola louti*) to an assemblage dominated by acanthurids. Around 1989, catches were correlated with increasing proportions of humphead wrasse (*C. undulatus*) and the large excavating parrotfish (*Chlorurus microrhinos*). In more recent years, the catch featured greater proportions of large browsing acanthurids *(Acanthurus xanthopterus* and *N. unicornis*) and one smaller bodied parrotfish (*Chlorurus sordidus*).

**Figure 4:**
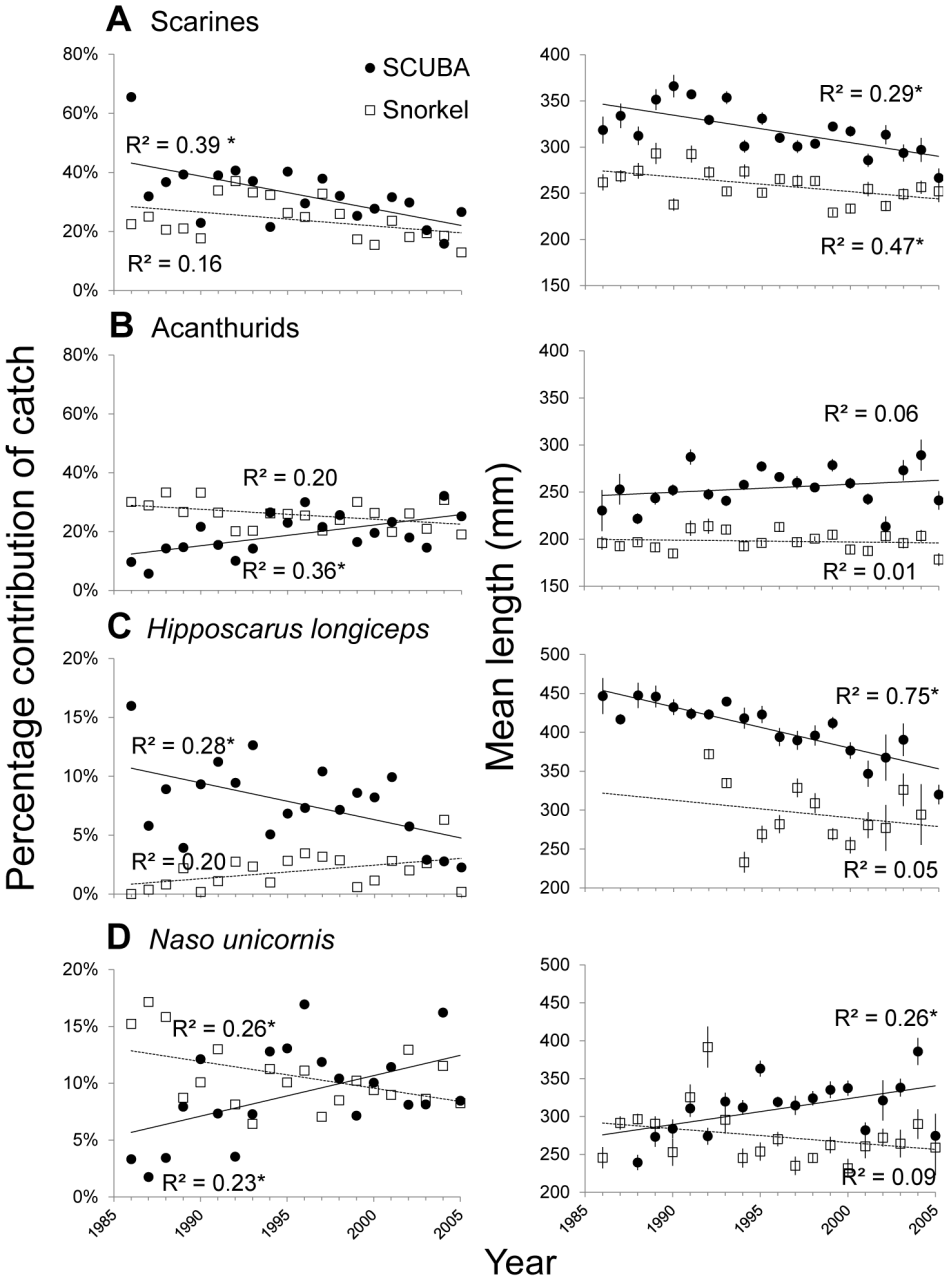
Percentage biomass contributions to catch and average (+ SE) mean length each year. Family groups; scarines (A) and acanthurids (B) and two frequently caught species; *Hipposcarus longiceps* (C) and *Naso unicornis* (D) caught by SCUBA spearfishing (black circles) and snorkel spearfishing (open squares). Significant regression values (p<0.05) indicated by asterisk (*).

The scarines, which dominated SCUBA spearfishing catch since the 1980s, experienced a significant decline in their percentage contribution to catch over time (Fig. 4A). Conversely, acanthurids became more common in catches during recent years (Fig. 4B). In contrast, the snorkel catch contribution of these fish groups has remained relatively consistent over time (Fig. 4A, 4B). This general pattern was also reflected in individual species within these groups. For example, the most heavily harvested parrotfish, *H. longiceps*, decreased in its contribution to SCUBA spearfishing catch over time (Fig. 4C), while *N. unicornis* decreased in snorkel spearfishing catch, but increased in the proportion of SCUBA spearfishing catch (Fig. 4D).

The mean lengths of scarines and acanthurids were greater when captured by SCUBA spearfishing compared to snorkel spearfishing (Fig. 4A, B). The average length of scarines decreased over time for both spearfishing methods, but there was no significant change in the length of acanthurids for either spearfishing method. While the mean harvested length of *H. longiceps* caught with SCUBA decreased significantly over time (Fig. 4C), the length of *N. unicornis* increased (Fig. 4D).

### Fishery-independent survey

#### Assemblage structure

In total, 6150 fish were counted from 210 stereo-BRUV replicates (135 fishery targeted species from 22 families) and 5712 of these fish were measured. Acanthurids and scarines were the most abundant fish groups recorded, together contributing to over half of all fish counted during this study, followed by wrasses (Labridae), goatfishes (Mullidae) and snappers (Lutjanidae).

**Figure 5:**
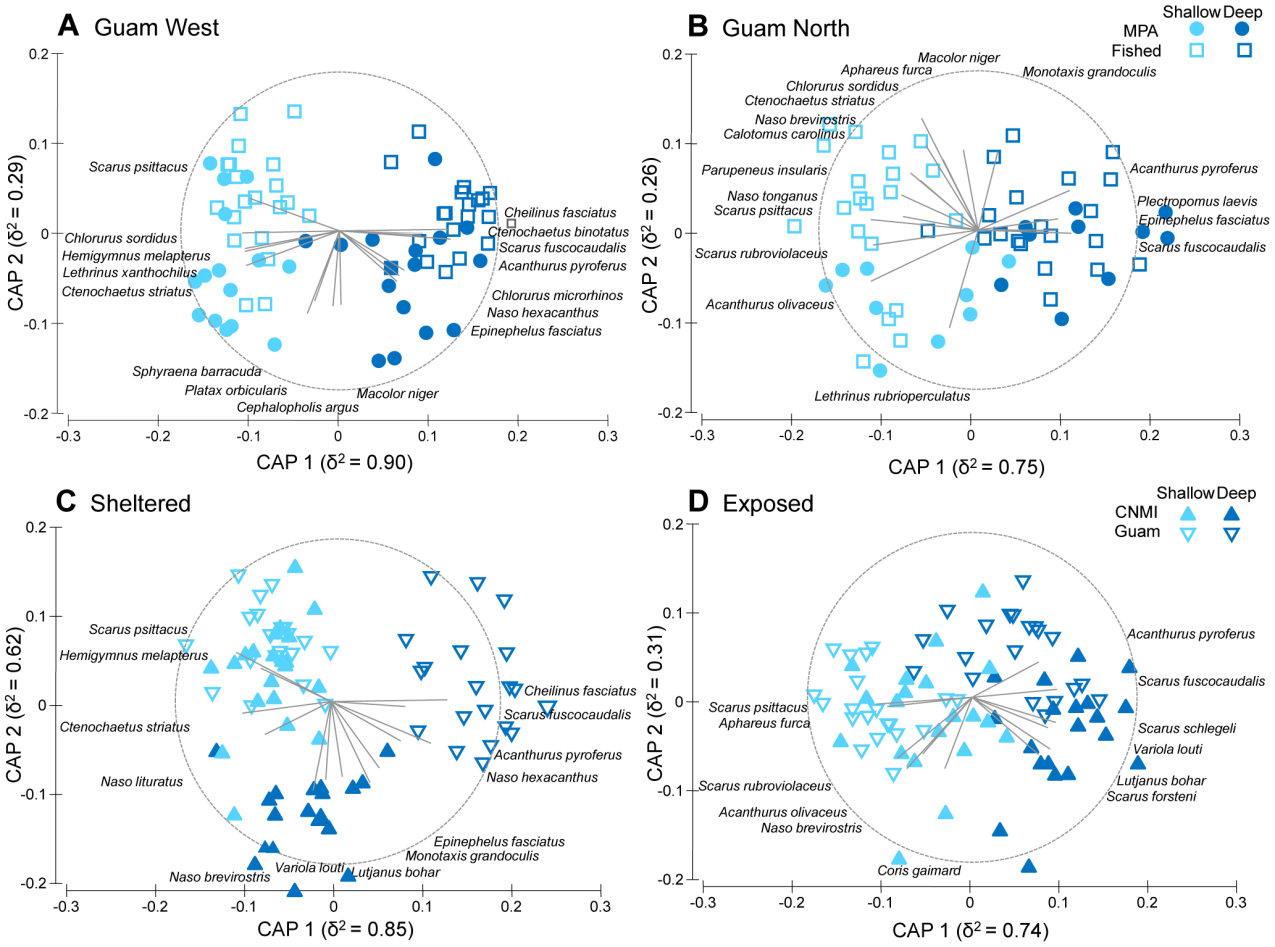
Canonical analysis of principal components (CAP) ordinations. Differences in assemblage structure of fished species biomass between MPA status and depth at each Guam location (A, B) and between jurisdiction and depth at each level of exposure (C, D). Species correlations with the canonical axis are indicated by the length and direction of vectors.

The assemblage of fished species consistently differed between depths at each location (p<0.01), but there were no differences between fished and MPA sites (Table S1). Between jurisdictions (Guam and CNMI) the significant interaction at the sheltered location (p<0.05) was further investigated with pairwise tests which showed that the fish assemblage was similar at shallow sites, but differed at the deep sites (t = 1.84, p = 0.03). The trace test statistic for canonical analysis of principal coordinates (CAP) was significant (p<0.001) for all comparisons indicating differences between depths and MPA status / jurisdiction (Fig. 5). The average cross validation allocation success ranged from 61–84% (Table S2), which was much higher than the allocation success rate of 25% which would be expected by chance with four groups. Canonical correlations (δ^2^) were highest (74–90%) on the first canonical axis (CAP 1) showing clear separation between depths, whereas lower correlations on the second canonical axis (26–29%) indicated less strength in the differences for MPA status (Fig. 5). The exception being between jurisdictions at the sheltered locations, where the second canonical axis (62%) showed clear separation between Guam and the CNMI in deep water but no difference in assemblage at the shallow depth (Fig. 5C). Several high value fish species were positively correlated with deep CNMI waters that are protected from SCUBA spearfishing; *Naso lituratus, Naso brevirostris*, *Variola louti*, and *Lutjanus bohar*.

#### Biomass of fished species and primary consumers

**Figure 6:**
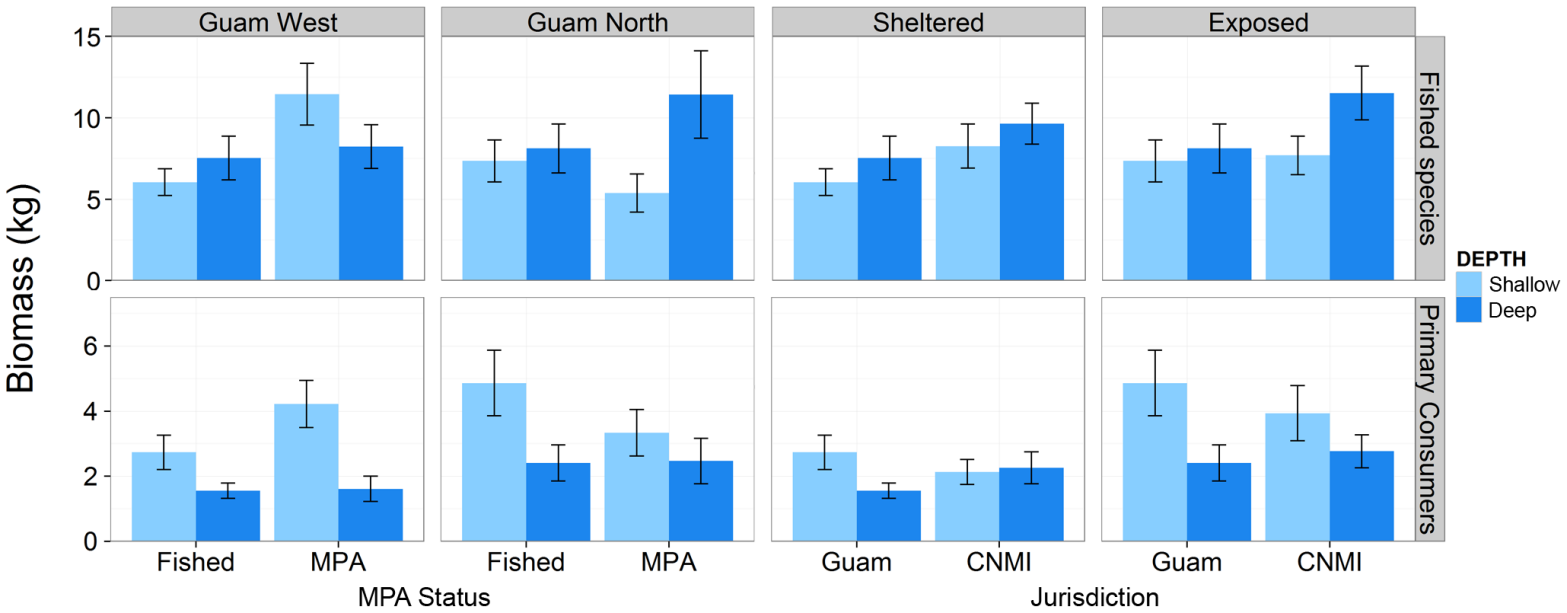
Mean biomass (± SE) of fished species (top row) and primary consumers (bottom row) at each depth. Comparisons are between fished and MPA sites at each Guam location (Guam West and Guam North) and between Guam and CNMI jurisdictions at each level of exposure (sheltered and exposed).

At Guam West, the mean biomass of fished species was greater within the MPA compared to fished sites (p<0.05, Table S3; Fig. 6). At the sheltered and exposed fished locations, there were no significant differences in biomass between jurisdictions or depths, though the highest biomass of fished species was found at the deep exposed location in the CNMI (Fig. 6). The greatest biomass of primary consumers was observed at the shallow exposed locations and the lowest biomass was found in the deep waters of Guam West (Fig. 6).

#### Lengths of scarines and acanthurids

**Figure 7:**
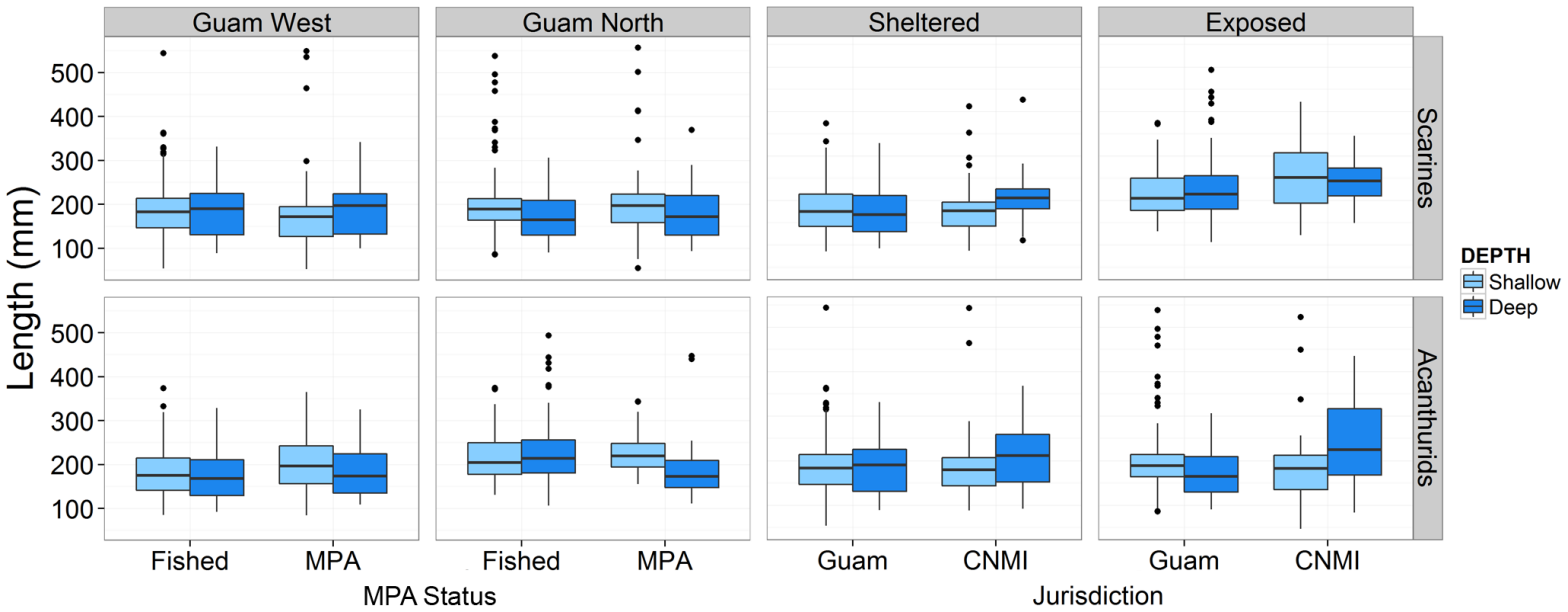
Boxplots of the lengths of scarines (top row) and acanthurids (bottom row) at each depth. Comparisons are between fished and MPA sites at each Guam location (Guam West and Guam North) and between Guam and CNMI jurisdictions at each level of exposure (sheltered and exposed).

At Guam West, parrotfishes (scarines) were slightly larger within the MPAs compared to fished sites and in shallow compared to deep sites (p = 0.053, Table S4; Fig. 7). At Guam North, the significant interaction term (p<0.001, Table S4) indicated scarine length was similar between MPA and fished sites at the shallow depth, but in deeper water, lengths were smaller within the MPA (t = 4.78, p<0.001, Fig. 7). Between jurisdictions at sheltered locations, the significant interaction term (p<0.05, Table S4) revealed similar lengths of scarines and acanthurids in shallow water, but lengths were both greater in deep waters at the CNMI compared to Guam (Scarines: t = 2.67, p<0.05, Acanthurids: t = 2.81, p<0.05; Fig.7). At exposed locations, scarines were larger at the CNMI compared to Guam at both depths (p<0.05, Table S4; Fig. 7b). The significant interaction for acanthurids (p<0.05, Table S4) revealed lengths were smaller in deeper water at Guam (t = 2.81, p<0.05, Fig. 7), in contrast to the CNMI where lengths were larger in deeper water (t = 2.57, p<0.05, Fig.7).

## Discussion

### Changes over time

There was a considerable change in SCUBA spearfishing catch composition over a 20 year period, shifting from a dominance of larger-bodied parrotfishes to a mixed assemblage with greater proportions of acanthurids. The shift from catches of large-bodied species, which tend to be highly vulnerable to fishing, to species with less vulnerable life histories is a widespread indicator of fisheries exploitation [49]. Increased targeting of herbivorous species by spearfishing has been documented elsewhere around the world after declining catches of larger piscivorous species [2], [50] or seasonal bans on grouper fishing [51], [52]. The lack of large species from higher trophic levels (e.g. piscivorous species such as grouper) in the Guam catches is likely associated with fishing impacts prior to the 1980s [14], [16]. However the decline in other high value species is exacerbated by SCUBA spearfishing, which targets large vulnerable species that sleep on the reef (e.g. *C. undulatus* and *H. longiceps*).

The reduced dominance of large-bodied parrotfishes and their declining average size is indicative of fisheries exploitation [53]–[55]. This can also affect the functional role of parrotfishes on coral reefs, since larger individuals can scrape and excavate much greater volumes of algal material than smaller fish [56], [57]. The micro-excavator *C. sordidus* was the only parrotfish to increase in the catch composition in recent years and was the most abundant parrotfish during our fishery-independent surveys. This supports claims that smaller parrotfish species are more resilient to fishing pressure than larger species and may support sustainable catches by spearfishers [53], [58], [59]. However, continued fishing for smaller species will also result in larger species being captured when encountered, thereby further increasing the risk of local extinctions of vulnerable species [58]. Species that comprised the majority of the SCUBA fishing catch in the 1980s, such as *S. rubroviolaceaus*, *C. microrhinos* and *S. forsteni*, were also found by Williams et al. [60] to be rare outside MPAs in Guam. Furthermore, biomass of these species was much greater at the unpopulated northern Mariana Islands [60], which suggests a depletion of these functionally important species in the southern Mariana Islands over the past two decades.

The large browsing herbivore *N. unicornis* contributed more to the spearfishing catch than any other acanthurid throughout the 20 years, with an increase in proportional contribution to SCUBA spearfishing catch and average length over time. Although the increased contributions to catch likely reflect fishers' shifting preference after the decline of other desirable species, this also poses the question how such a heavily fished species can show signs of resilience to fishing pressure. A recent study showed high genetic diversity of adults and recruit *N. unicornis* on Guam, which suggests significant larval mixing and migrant exchange [61]. It is therefore possible that recruitment is decoupled from the adult reproductive stock, such that replenishment occurs from outside the local population. Apart from direct evidence of connectivity with Saipan, the upper limit for migrant exchange for this species is beyond the scale of the Mariana Islands and could encompass other Micronesian islands, especially those to the south-east from which the North Equatorial Current flows [61], [62]. This level of connectivity stands in contrast to another heavily fished species, *Siganus spinus*, which was found to have a high level of self-recruitment within the Mariana Islands [62]. *Naso unicornis* is regarded as a highly important food-fish species in Micronesia [9], [11] and plays an important role in the removal of macroalgae on coral reefs [13]. Therefore, and despite the fact that it does not show typical responses indicating overexploitation, protection is warranted to ensure that commercial harvesting does not limit the availability of this species for shallow water subsistence fishing and performing key ecological functions.

### Refuge from fishing pressure

Catch data clearly showed that SCUBA spearfishing captures larger fish than snorkel spearfishing. Although the capture of large individuals is often regarded as good practice by fishers as they are not harvesting immature fish, heavy fishing of larger-bodied individuals can also have a substantial impact by removing a population's spawning biomass [63], [64]. Population models revealed that protection in the form of MPAs allowed larger and older individuals of the highly exploited reef fish *Lethrinus harak* to increase in number, yielding considerable reproductive benefits in Guam [65]. Deeper waters that are inaccessible to certain fishing methods could provide protection to reef fish in the same way as spatial closures by allowing spillover of adults or recruits. This was exemplified in the Californian abalone fishery, where a ban on the use of SCUBA allowed a greater biomass of legal-sized abalone to accumulate in deeper water, providing localised recruitment over time and supporting high catch yields in shallower waters [28]. In locations where a ban on SCUBA was not implemented, low numbers of reproductively active individuals across the depth range resulted in a collapse of the fishery [28]. As SCUBA spearfishers can access deep-water reefs and selectively target larger individuals, the use of this fishing method may limit the reproductive benefits from remaining populations that have been overexploited at shallow depths.

Depth refuge from fishing pressure may be a widespread effect, especially for tropical artisanal fisheries that primarily use gears such as spear guns and nets to target shallow water populations [8], [27]. Previous studies focused on depth refuge for coral reef fish have based their conclusions on differences in species richness and the presence/absence of certain species [26], [27]. Our study expands on this by using two classic fishery indicators, biomass and length, in addition to changes in assemblage structure. While there is some evidence that SCUBA spearfishing is still practiced in the CNMI (albeit at a reduced level; [9]), we found lengths of scarines and acanthurids to be of a greater size in deeper waters of CNMI compared to Guam. Similarly, several fished species were positively correlated with this deeper refuge at deep sites where SCUBA spearfishing is banned, while there was little difference in assemblage structure in shallow waters where snorkel spearfishing is practiced. It is apparent from these results that deeper waters provide refuge from fishing impacts when protected from deep water fishing methods such as SCUBA spearfishing (Fig. S1).

While MPAs provide refuge from fishing pressure, exposure may also play a role by limiting access to fishers during periods of rough weather. This is likely prevalent in our study, where exposed sites were also located far from boat ramps. Although our results support those of Mumby et al. [34], who also found greater biomass of herbivores in exposed locations, the latter study suggests this is primarily due to high wave exposure increasing primary productivity and hence food resources for herbivores, rather than the effects of limiting fishing pressure. Although there was some indication of fish assemblages at deep sites differing inside and outside MPAs at Guam West, we did not observe other positive deep water MPA effects for biomass and length. This may be due to the small size of the protected area or the potential for poaching at night using SCUBA. Accordingly, Goetze et al. [26] only detected depth refuge for species richness in a large, well established MPA (over three times the size of the Guam North MPA) and no difference in a small, newly established reserve (similar in size to the Guam West MPA). The coral reefs of Guam were heavily fished prior to the establishment of the MPAs [15], [16], hence it is likely that MPAs in Guam are still recovering and will continue to increase in fish biomass well after the current 10+ years of protection [66], [67]. Although we did not observe MPAs on Guam to show positive effects from the protection of SCUBA spearfishing in deep water, continued monitoring is recommended as these areas were associated with increased biomass of fish in sheltered shallow waters.

### Species of concern

SCUBA spearfishing is associated with the capture of large species of high conservation concern. Four of the species caught in greater proportions by SCUBA spearfishing compared to snorkel spearfishing have been assessed by the International Union for the Conservation of Nature (IUCN) and are classified as either endangered (*C. undulatus*), vulnerable (*B. muricatum* and *P. laevis*) or near threatened (*E. polyphekadion*) [68]. For example, 95% of the spearfishing catches of humphead wrasse (*C. undulatus*) were caught using SCUBA. Sadovy et al. (2003) inform that the decline of the humphead wrasse and its subsequent listing as endangered is attributed to overfishing. However, this large iconic species is also highly valuable when kept alive for dive tourism [69], [70] which provides a much greater revenue to Guam than the commercial fishing industry [71]. Depth was found to be the strongest predictor of this species' distribution in the Mariana Archipelago [72], which was supported by our own observations of 15 *C. undulatus* individuals, of which 80% were found at the deeper depth (30 m). A restriction on SCUBA spearfishing would ensure critical refuge habitat in deeper water and the potential for recovery of this endangered species.

The giant bumphead parrotfish (*B. muricatum*) is a keystone species in the regulation of reef growth and another species of particular conservation concern [73]. Their large size make them a valuable catch for island communities, while their habit of sleeping on the reef in groups make them highly susceptible to night-time spearfishing [74], [75]. During the 1980s, fishing for *B. muricatum* on Guam took place at night using SCUBA with the subsequent catch sold directly to hotels in the early morning, and was largely underreported by creel surveys (G. Davis, personal communication). Our fishery-independent surveys did not detect a single *B. muricatum* in the southern Mariana Islands, a finding consistent with other studies that have collectively surveyed virtually the entire length of Guam's coastline [34], [60], [72], [76]. While large schools of over one hundred *B. muricatum* were commonly observed around Guam before the introduction of SCUBA spearfishing in the late 1970s (G. Davis, personal communication), now both adult fish and new recruits are rarely, if ever, observed. With possible localised extinction of the adult population, recruitment will be significantly reduced, especially under a scenario of a coupled stock-recruitment relationship.

### Management recommendations

Our analyses of the catch data clearly demonstrated that SCUBA spearfishing has had a long-term and ongoing impact on reef fish communities in Guam, particularly affecting large vulnerable species. Impacts were likely exacerbated by factors such as fishing at night, access to commercial markets and the lack of catch quotas, size limits and protection for certain species. Restriction or management of any of these factors could reduce the severity of fishing impacts (as suggested by Houk et. al [9]). However, even when management regulations apply, such as in Australia, spearfishing can still have rapid and substantial negative effects on fish populations [4] and anecdotal evidence suggests that SCUBA spearfishing did have a serious impact on near-shore fish communities during the 1970s [77]. Therefore, in countries around the world where restrictions on SCUBA spearfishing have not been established, we recommend simple gear-based restrictions. Experience in other countries shows that a general ban on the use of SCUBA for spearfishing is often insufficient because of difficulties in obtaining evidence for court prosecutions that fish were taken when SCUBA diving [1]. New legislation should therefore create an offence for possessing SCUBA gear and fishing gear in the same boat or car (as recommended by Gillet and Moy [1]). A ban on this fishing method has been recommend by various authors to ensue more sustainable reef fish catches [1], [2], [14], [15], [77]. It has also been noted that the residents of Guam generally support a ban on night-time SCUBA spearfishing [71].

Gear-based restrictions, although more easily enforced than multispecies catch limits, can have unintended consequences such as the displacement of fishing effort. Even though a ban on SCUBA fishing in American Samoa was successful in protecting vulnerable fishery resources, the fishery did not completely cease and was instead displaced to the neighboring island of Samoa [1], [17]. This shifting effort is of particular concern, especially in Micronesia where reef fish imports to Guam are increasing, yet remain unregulated and unreported [3], [15]. Although not frequently practiced, SCUBA spearfishing also remains legal in the nearby islands of Yap, Chuuk and the Marshall Islands. Yap is one of few islands where large vulnerable species such as *C. undulatus* and *B. muricatum* are still regularly caught for local markets [9]. With plans to develop a large tourism industry in Yap, there is concern that without introducing precautionary fishery management approaches, the boom in tourism and resulting changes in economy will increase fishing pressure to unsustainable levels. Lessons must be learnt from Guam's experience in the 1980s, when a rapid increase in tourism and associated demand for reef fish encouraged commercial snorkel spearfishing at night. Soon after, catch rates declined from the shallow waters and fishers resorted to using SCUBA to access deeper waters in more remote locations [78]. Since the management of established fisheries via a top-down approach is more difficult because stakeholder compliance is often low [79], [80], we suggest a-priori restrictions on SCUBA spearfishing for communities where the fishery has not yet commenced but has the potential to develop.

## Supporting Information

Figure S1 **Illustration of the difference between snorkel and SCUBA spearfishing and the potential for depth refuge.**
(PDF)

Table S1 **Three-way PERMANOVA testing for differences in the assemblage of targeted species.** Comparisons are between MPA status and depth at Guam locations, and between jurisdiction and depth at sheltered and exposed sites. Significant p values (<0.05) are shown in bold.
(DOCX)

Table S2 **CAP leave-one-out allocation of observations to groups.**
(DOCX)

Table S3 **ANOVAs examining the biomass of fished species and primary consumers.** Comparisons are between MPA status and depth at the two Guam locations and between jurisdiction and depth, at sheltered and exposed sites. Significant p values (<0.05) are shown in bold.
(DOCX)

Table S4 **ANOVAs examining the lengths of scarines and acanthurids.** Comparisons are between factors MPA status and depth at the two Guam locations and between jurisdiction and depth, at sheltered and exposed sites. Significant p values (<0.05) are shown in bold.
(DOCX)
