# Supplementary figures and images for: Depth Refuge and the Impacts of SCUBA Spearfishing on Coral Reef Fishes
Source: PLoS One. 2014 Mar 24;9(3):e92628. doi: 10.1371/journal.pone.0092628 (PMC3963921; doi:10.1371/journal.pone.0092628)

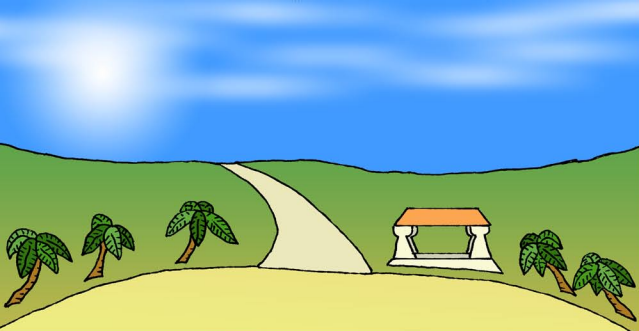

## Depth Refuge

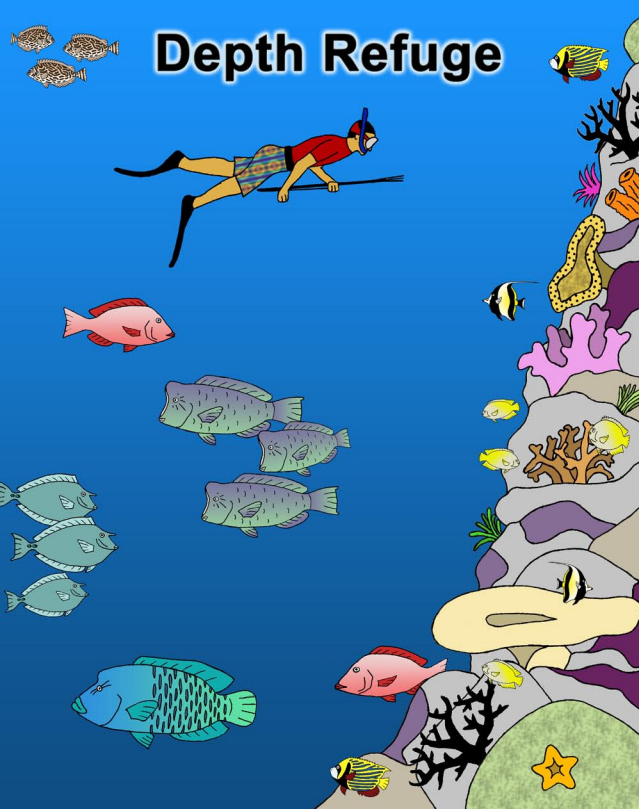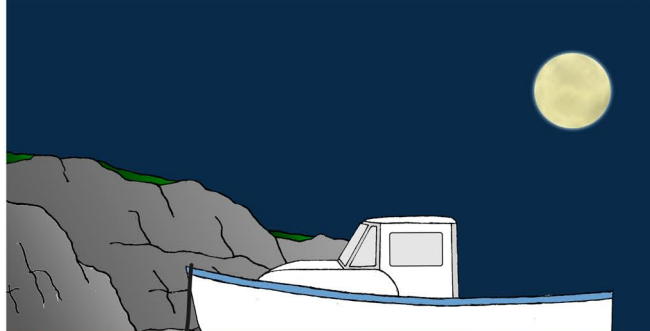

## No Depth Refuge

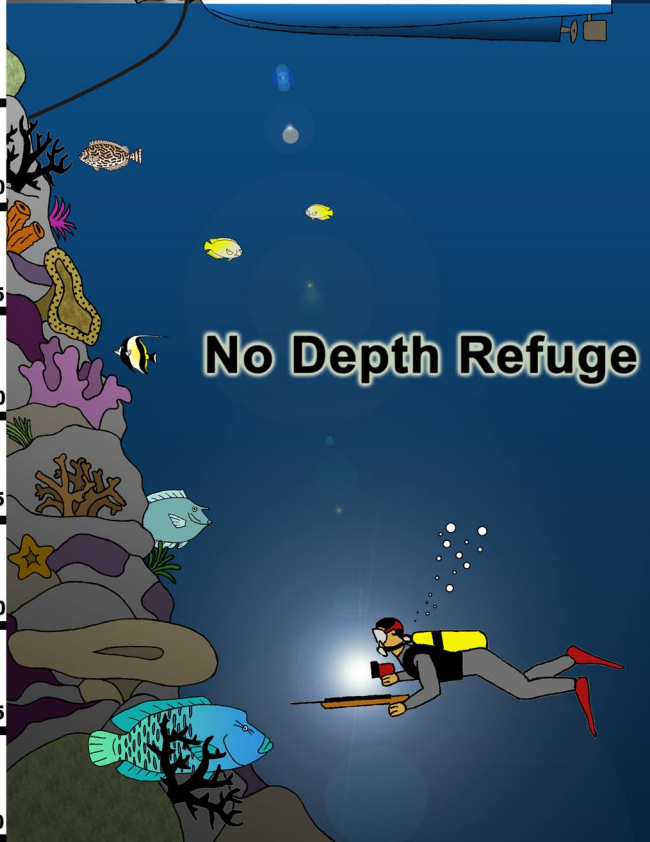

Supplement: Figure S1 — Illustration of the difference between snorkel and SCUBA spearfishing and the potential for depth refuge. (PDF) [file pone.0092628.s001.pdf]
