# Supplementary material for: Depth Refuge and the Impacts of SCUBA Spearfishing on Coral Reef Fishes
Source: PLoS One. 2014 Mar 24;9(3):e92628. doi: 10.1371/journal.pone.0092628 (PMC3963921; doi:10.1371/journal.pone.0092628)
Supplement: Table S1 — Three-way PERMANOVA testing for differences in the assemblage of targeted species. Comparisons are between MPA status and depth at Guam locations, and between jurisdiction and depth at sheltered and exposed sites. Significant p values (<0.05) are shown in bold. (DOCX) [file pone.0092628.s002.docx]

**Table S1: Three-way PERMANOVA testing for differences in the assemblage of targeted species**. Comparisons are between MPA status and depth at Guam locations, and between jurisdiction and depth at sheltered and exposed sites. Significant p values (< 0.05) are shown in bold.

|  | *Guam West* | | | | *Guam North* | | | |
| --- | --- | --- | --- | --- | --- | --- | --- | --- |
| **Source** | ***df*** | ***MS*** | ***F*** | ***P*** | ***df*** | ***MS*** | ***F*** | ***P*** |
| MPA Status | 1 | 5.942 | 1.132 | 0.302 | 1 | 6.280 | 1.149 | 0.306 |
| Depth | 1 | 32.250 | 6.145 | **0.001** | 1 | 19.842 | 3.629 | **0.002** |
| ST x DE | 1 | 4.374 | 0.833 | 0.682 | 1 | 5.066 | 0.926 | 0.540 |
| Site (ST x DE) | 10 | 5.248 | 1.658 | **<0.001** | 8 | 5.468 | 1.461 | **<0.001** |
| Residual | 56 | 7.277 |  |  | 48 | 3.743 |  |  |
|  | *Sheltered* | | | | *Exposed* | | | |
| Jurisdiction | 1 | 13.111 | 2.286 | **0.014** | 1 | 9.581 | 1.632 | 0.052 |
| Depth | 1 | 23.029 | 4.016 | **0.001** | 1 | 22.767 | 3.878 | **<0.001** |
| JU x DE | 1 | 10.740 | 1.873 | **0.042** | 1 | 5.414 | 0.922 | 0.534 |
| Site (JU x DE) | 12 | 5.735 | 1.822 | **<0.001** | 12 | 5.872 | 1.562 | **<0.001** |
| Residual | 64 | 3.148 |  |  | 64 | 3.758 |  |  |
