# Supplementary material for: Depth Refuge and the Impacts of SCUBA Spearfishing on Coral Reef Fishes
Source: PLoS One. 2014 Mar 24;9(3):e92628. doi: 10.1371/journal.pone.0092628 (PMC3963921; doi:10.1371/journal.pone.0092628)
Supplement: Table S2 — CAP leave-one-out allocation of observations to groups. (DOCX) [file pone.0092628.s003.docx]

**Table S2: CAP leave-one-out allocation of observations to groups**

|  | | *Guam West (m=11)* | | | | | *Guam North (m=5)* | | | | | |
| --- | --- | --- | --- | --- | --- | --- | --- | --- | --- | --- | --- | --- |
| **Original group** | | **Fished** | | **MPA** | |  | **Fished** | | | **MPA** | |  |
|  | | **D** | **S** | **D** | **S** | **% correct** | **D** | **S** | | **D** | **S** | **% correct** |
| **Fished** | **D** | 15 | 0 | 5 | 0 | 75 | 13 | 0 | | 6 | 1 | 65 |
| **Fished** | **S** | 0 | 14 | 0 | 6 | 70 | 1 | 15 | | 0 | 4 | 75 |
| **MPA** | **D** | 4 | 2 | 9 | 0 | 60 | 5 | 0 | | 4 | 1 | 40 |
| **MPA** | **S** | 0 | 5 | 1 | 9 | 60 | 2 | 3 | | 0 | 5 | 50 |
|  | | *Sheltered (m=7)* | | | | | *Exposed (m=9)* | | | | | |
| **Original group** | | **Guam** | | **CNMI** | |  | **Guam** | | | **CNMI** | |  |
|  | | **D** | **S** | **D** | **S** | **% correct** | **D** | **S** | | **D** | **S** | **% correct** |
| **Guam** | **D** | 20 | 0 | 0 | 0 | 100 | 12 | | 0 | 5 | 3 | 60 |
| **Guam** | **S** | 0 | 17 | 0 | 3 | 85 | 0 | | 14 | 0 | 6 | 70 |
| **CNMI** | **D** | 1 | 1 | 17 | 1 | 85 | 5 | | 0 | 14 | 1 | 70 |
| **CNMI** | **S** | 0 | 4 | 3 | 13 | 65 | 3 | | 6 | 2 | 9 | 45 |
