# Supplementary material for: Depth Refuge and the Impacts of SCUBA Spearfishing on Coral Reef Fishes
Source: PLoS One. 2014 Mar 24;9(3):e92628. doi: 10.1371/journal.pone.0092628 (PMC3963921; doi:10.1371/journal.pone.0092628)
Supplement: Table S3 — ANOVAs examining the biomass of fished species and primary consumers. Comparisons are between MPA status and depth at the two Guam locations and between jurisdiction and depth, at sheltered and exposed sites. Significant p values (<0.05) are shown in bold. (DOCX) [file pone.0092628.s004.docx]

**Table S3: ANOVAs examining the biomass of fished species and primary consumers.** Comparisons are between MPA status and depth at the two Guam locations and between jurisdiction and depth, at sheltered and exposed sites. Significant p values (< 0.05) are shown in bold.

|  |  | *Guam West* | | | | | | *Guam North* | | | |  |
| --- | --- | --- | --- | --- | --- | --- | --- | --- | --- | --- | --- | --- |
|  |  | ***df*** | ***MS*** | ***F*** | ***P*** | | ***df*** | | ***MS*** | ***F*** | ***P*** | |
| **Fished** | MPA status | 1 | 159.97 | 5.1 | **0.048** | | 1 | | 5.94 | 0.07 | 0.794 | |
| **species** | Depth | 1 | 13.01 | 0.41 | 0.534 | | 1 | | 155.44 | 1.92 | 0.204 | |
|  | ST x DE | 1 | 95.25 | 3.03 | 0.112 | | 1 | | 93.17 | 1.15 | 0.315 | |
|  | Site (ST x DE) | 10 | 31.38 | 1 | 0.458 | | 8 | | 81.11 | 2.42 | **0.028** | |
|  | Error | 56 | 31.5 |  |  | | 48 | | 33.51 |  |  | |
| **Primary** | MPA status | 1 | 0.5295 | 3.43 | 0.094 | | 1 | | 7.24 | 0.24 | 0.64 | |
| **consumers** | Depth | 1 | 4.5904 | 29.75 | **<0.001** | | 1 | | 36.6 | 1.19 | 0.307 | |
|  | ST x DE | 1 | 0.6897 | 4.47 | 0.061 | | 1 | | 8.42 | 0.27 | 0.615 | |
|  | Site (ST x DE) | 10 | 0.1543 | 0.6 | 0.805 | | 8 | | 30.68 | 4.22 | **<0.001** | |
|  | Error | 56 | 0.2563 |  |  | | 48 | | 7.28 |  |  | |
|  |  | *Sheltered* | | | | *Exposed* | | | | | |  |
| **Fished** | Jurisdiction | 1 | 93.77 | 2.16 | 0.167 | | 1 | | 70.2 | 1.19 | 0.296 | |
| **species** | Depth | 1 | 41.03 | 0.94 | 0.35 | | 1 | | 106.1 | 1.8 | 0.204 | |
|  | JU x DE | 1 | 0.06 | 0 | 0.972 | | 1 | | 46.96 | 0.8 | 0.389 | |
|  | Site (JU x DE) | 12 | 43.42 | 1.61 | 0.112 | | 12 | | 58.86 | 1.61 | 0.112 | |
|  | Error | 64 | 27.02 |  |  | | 64 | | 36.65 |  |  | |
| **Primary** | Jurisdiction | 1 | 0 | 0 | 0.972 | | 1 | | 0.02 | 0.02 | 0.881 | |
| **consumers** | Depth | 1 | 0.38 | 1.53 | 0.239 | | 1 | | 2.81 | 2.76 | 0.123 | |
|  | JU x DE | 1 | 0.49 | 2.01 | 0.182 | | 1 | | 0.46 | 0.45 | 0.516 | |
|  | Site (JU x DE) | 12 | 0.25 | 1.11 | 0.37 | | 12 | | 1.02 | 2.37 | **0.014** | |
|  | Error | 64 | 0.22 |  |  | | 64 | | 0.43 |  |  | |
