# Supplementary material for: Depth Refuge and the Impacts of SCUBA Spearfishing on Coral Reef Fishes
Source: PLoS One. 2014 Mar 24;9(3):e92628. doi: 10.1371/journal.pone.0092628 (PMC3963921; doi:10.1371/journal.pone.0092628)
Supplement: Table S4 — ANOVAs examining the lengths of scarines and acanthurids. Comparisons are between factors MPA status and depth at the two Guam locations and between jurisdiction and depth, at sheltered and exposed sites. Significant p values (<0.05) are shown in bold. (DOCX) [file pone.0092628.s005.docx]

**Table S4: ANOVAs examining the lengths of scarines and acanthurids.** Comparisons are between factors MPA status and depth at the two Guam locations and between jurisdiction and depth, at sheltered and exposed sites. Significant p values (< 0.05) are shown in bold.

|  |  | *Guam West* | | | | | *Guam North* | | | | |
| --- | --- | --- | --- | --- | --- | --- | --- | --- | --- | --- | --- |
|  |  | ***df*** | ***MS*** | ***F*** | ***P*** | | ***df*** | | ***MS*** | ***F*** | ***P*** |
| **Scarines** | MPA status | 1 | 12651 | 3.75 | 0.053 | | 1 | | 15091 | 4.38 | **0.037** |
|  | Depth | 1 | 12726 | 3.77 | 0.053 | | 1 | | 24317 | 7.05 | **0.008** |
|  | ST x DE | 1 | 457 | 0.14 | 0.713 | | 1 | | 77761 | 22.56 | **<0.001** |
|  | Error | 377 | 3372 |  |  | | 238 | | 3447 |  |  |
| **Acanthurids** | MPA status | 1 | 1018 | 0.26 | 0.611 | | 1 | | 1958 | 0.38 | 0.536 |
|  | Depth | 1 | 1339 | 0.34 | 0.56 | | 1 | | 40461 | 7.92 | **0.005** |
|  | ST x DE | 1 | 6146 | 1.56 | 0.212 | | 1 | | 3517 | 0.69 | 0.407 |
|  | Error | 396 | 3928 |  |  | | 315 | | 5106 |  |  |
|  |  | *Sheltered* | | | | | *Exposed* | | | | |
| **Scarines** | Jurisdiction | 1 | 3109 | 1.07 | | 0.302 | 1 | 16935 | | 4.04 | **0.046** |
|  | Depth | 1 | 5609 | 1.93 | | 0.166 | 1 | 4758 | | 1.13 | 0.288 |
|  | JU x DE | 1 | 24334 | 8.37 | | **0.004** | 1 | 3389 | | 0.81 | 0.37 |
|  | Error | 314 | 2908 |  | |  | 314 | 4197 | |  |  |
| **Acanthurids** | Jurisdiction | 1 | 10895 | 2.64 | | 0.105 | 1 | 63891 | | 11.13 | **0.001** |
|  | Depth | 1 | 8739 | 2.12 | | 0.147 | 1 | 19166 | | 3.34 | 0.069 |
|  | JU x DE | 1 | 18269 | 4.42 | | **0.036** | 1 | 174639 | | 30.42 | **<0.001** |
|  | Error | 394 | 4130 |  | |  | 355 | 5740 | |  |  |
